# Effects of metazachlor and its major metabolite metazachlor OA on early life stages of marbled crayfish

**DOI:** 10.1038/s41598-020-57740-1

**Published:** 2020-01-21

**Authors:** Josef Velisek, Alzbeta Stara, Jan Kubec, Eliska Zuskova, Milos Buric, Antonin Kouba

**Affiliations:** University of South Bohemia in Ceske Budejovice, Faculty of Fisheries and Protection of Waters, South Bohemian Research Center of Aquaculture and Biodiversity of Hydrocenoses, Research Institute of Fish Culture and Hydrobiology, Zatisi 728/II, 389 25 Vodnany, Czech Republic

**Keywords:** Metabolomics, Environmental impact

## Abstract

The effects of the herbicide metazachlor and its major metabolite metazachlor OA at two concentrations, including environmentally relevant concentrations of metazachlor (0.0115 µmol/l and 0.0790 µmol/l) and metazachlor OA (0.0117 µmol/l and 0.0805 µmol/l), respectively, were evaluated on early ontogeny, growth, behaviour, oxidative stress, antioxidant enzyme levels, histology, and mortality of marbled crayfish *Procambarus virginalis*. Both tested concentrations of metazachlor and metazachlor OA were associated with significantly lower growth and delayed ontogenetic development compared to controls. Exposure of metazachlor at 0.0115 µmol/l and metazachlor OA at 0.0117 µmol/l and 0.0805 µmol/l resulted in significantly lower activity of total superoxide dismutase (SOD), catalase (CAT), glutathione s-transferase (GST), glutathione reductase (GR), and reduced glutathione (GSH) compared with control and resulted in gill anomalies ranging from wall thinning to focal disintegration of branchial structure. Metazachlor at the environmentally relevant concentration of 0.0790 µmol/l was associated with significant alterations of crayfish distance moved and walking speed. The potential risk associated with metazachlor use in agriculture related to effects on non-target aquatic organisms.

## Introduction

Pollution of ecosystems is a serious problem worldwide. Herbicides are of frequent pollutants in surface and groundwaters with highest concentrations observed during runoff and after agricultural applications^1–3^.

Metazachlor (2-chloro-N-(2,6-dimethylphenyl)-N-(1H-pyrazol-1-ylmethyl)-acetamide) is herbicide used on oil seed crops. Metazachlor is a member of the chloroacetamide class of chemicals, which inhibit the formation of long chain fatty acids that play a key role in cell division and cell expansion processes^4^. Metazachlor is used for pre-emergence and early post-emergence control of annual grasses and broadleaf weeds in crops^5^. Metazachlor was evaluated for FAO specifications under the procedure adopted in 1999^6^.

Metazachlor is reported to be moderately toxic to fish^7^. The 96hLC50 values of metazachlor determined for rainbow trout (*Oncorhynchus mykiss*) is 4.0 mg/l, for bluegill sunfish (*Lepomis macrochirus*) 15.0 mg/l, and, for common carp (*Cyprinus carpio*), 15.0 mg/l^6^. The green alga (*Pseudokirchneriella subcapitata*) is highly sensitive to metazachlor with 72hEC50 of 0.031 mg/l^6^, whereas green alga *Chlorella* spp. are insensitive with 96hEC50 of 1.63 mg/l^8^. A 48hEC50 value of 22.3 mg/l was found for *Daphnia magna*^6^. The herbicide Butisan 400 SC containing 35.6% metazachlor applied at 5.0 mg/l induced vitellogenin synthesis in zebrafish (*Danio rerio*)^9^. Metazachlor has pronounced effects on aquatic macrophytes and aquatic ecosystem function at concentrations exceeding 5.0 µg/l^10,11^. In mammals, carcinogenic effects have been reported in addition to acute toxicity^12^. While effects of metazachlor exposure on vertebrates have been well documented, there is no data with respect to the impact of metazachlor and its metabolites on crustaceans.

The major degradation products of metazachlor in water are metazachlor ESA [N-(2,6-dimethylphenyl)-N-(1H-pyrazol-1-ylmethyl)aminocarbonylmethylsulfonic acid] and metazachlor OA [N-(2,6-dimethylphenyl)-N-(1H-pyrazol-1-ylmethyl)oxalamide]. Metazachlor has a half-life in soils of 5–30 days, degrading to oxanilic acid (OA), ethane sulfonic acid (ESA), and derivatives^13,14^. Metazachlor is stable to aqueous hydrolysis at temperatures and pH levels. The transformation products are only weakly adsorbed into soil, resulting in mobility. The metabolites of metazachlor are among the common pollutants^13,15,16^. Metazachlor has been reported in waters at levels 0.1 μg/l −100 μg/l^3,10,11,17–19^. Metazachlor OA and metazachlor ESA have been found in surface water at concentrations as high as 1.8 and 4.8 µg/l, respectively^19^. The maximum level of metazachlor and metazachlor OA detected in Czech rivers is 22 µg/l and 3.2 µg/l, respectively^20^. Lazartigues *et al*.^21^ found a metazachlor level of 0.13 ± 0.02 μg/kg ww in muscle of European perch *Perca fluviatilis* from a pond in north-eastern France. Metazachlor and its metabolites pose a potential risk to exposed aquatic communities.

The present study were to determine effects of environmentally relevant concentrations of metazachlor and its major metabolite, metazachlor OA, on the marbled crayfish. Objectives were to substantiate the impacts of exposure on mortality, growth, ontogenetic development, oxidative stress, antioxidant biomarkers, behaviour, and histology.

## Methods

### Chemicals and chemical analysis

Metazachlor (purity 99.7%) was purchased from Sigma-Aldrich Corporation (USA) and metazachlor OA (purity 98.7%) from Neochema GmbH (Germany). To ensure agreement between nominal and actual metazachlor and metazachlor OA concentrations in water, samples were analysed by liquid chromatography tandem mass spectrometry (LC-MS/MS) according to Ramos *et al*.^22^. In the water samples, the limit of quantification (LOQ) of metazachlor and metazachlor OA was 0.01 µg/l. The concentrations of metazachlor and metazachlor OA in the de-chlorinated tap water and in control crayfish during the trial were below the LOQ. The concentration of metazachlor and metazachlor OA in water over the course of 48 h as mean of the six sampling dates compared to the nominal value for all test groups is presented in Table 1.

**Table 1:** Concentration of metazachlor and metazachlor OA in water in test of toxicity to early-life stages of marbled crayfish.

Group	Sampling time (h)	Concentration (µmol/l)
Tap water	—	<LOQ
Control	0	<LOQ
	24	<LOQ
	48	<LOQ
M1 - Metazachlor (0.0115 µmol/l)	0	0.0110 ± 0.003
	24	0.0103 ± 0.004
	48	0.0101 ± 0.005
M2- Metazachlor (0.0790 µmol/l)	0	0.0798 ± 0.007
	24	0.0788 ± 0.004
	48	0.0775 ± 0.005
MOA1 - Metazachlor OA (0.0117 µmol/l)	0	0.0119 ± 0.002
	24	0.0115 ± 0.001
	48	0.0115 ± 0.003
MOA2 - Metazachlor OA (0.0805 µmol/l)	0	0.0807 ± 0.010
	24	0.0790 ± 0.009
	48	0.0770 ± 0.008

The concentrations were measured immediately after water exchange (0 h), 24 h, and 48 h post-exchange. The values are expressed as mean ± SD, n = 6. LOQ = limit of quantification.

### Experimental animals

Parthenogenetic juveniles at stage 3 of embryonic development (mean weight of 5.31 ± 0.22 mg), offspring of a single marbled crayfish female (carapace length 24.50 mm, postorbital carapace length 28.36 mm, weight 6.4 g), were used. All the methods used in the present study followed relevant guidelines and regulations. Also, the competent authority (Ethical Committee for the Protection of Animals in Research of the University of South Bohemia, FFPW Vodnany) approved the experiment and protocols of the present study.

### Experimental protocol

Two hundred juvenile crayfish were placed in individual 20 ml plastic macroplates. Two concentrations of metazachlor, two concentrations of metazachlor OA, and a control were used, 40 specimens per group. The tested concentrations were

M1 – metazachlor at 0.0115 µmol/l ( = 3.2 µg/l)

M2 – metazachlor at 0.0790 µmol/l ( = 22 µg/l - maximum reported concentration in a Czech river^20^)

MOA1 – metazachlor OA at 0.0117 µmol/l ( = 3.2 µg/l – max. reported concentration in a Czech river^20^)

MOA2 – metazachlor OA at 0.0805 µmol/l ( = 22 µg/l)

Treatment without tested compounds (tap water) served as control (C). The macroplates were maintained under a 12:12 h light:dark regime. The test was semistatic, with solutions renewed three times weekly (Mon, Wed, Fri). Water quality parameters were temperature 21.51 ± 0.71 °C, dissolved oxygen saturation >89.75%, pH 7.74–8.01. Temperature was measured hourly using Minikin loggers (Environmental Measuring Systems, Brno, Czech Republic). Crayfish were fed *ad libitum* on freshly hatched brine shrimp *Artemia salina* nauplii. Animals were monitored daily for mortality, ontogenetic development, morphological anomalies, and body weight at developmental stages (second day after moulting to allow at least partial calcification of the exoskeleton). Definition of developmental stages followed Vogt *et al*.^23^.

The toxicity test was terminated after 40 days, at which time crayfish behaviour was recorded. After recording, all survive crayfish were sacrificed on ice anaesthesia, weighed, and stored at −80 °C until further processing.

### Growth rate

After removing excess water by filter paper, body weight was determined using a Mettler-Toledo (Greifensee, Switzerland) analytical balance to the nearest 0.1 mg. The mean specific growth rates (SGR) of experimental groups were calculated for the period from the first sampling time (day 5) to last sampling time end of the trial (day 40). The SGR was individually calculated for all crayfish surviving at the end of test. Inhibition of growth of exposed groups compared with control was calculated using the method described by Organization for Economic Cooperation and Development^24^.

### Locomotion

At the end of the test, surviving crayfish were recorded using simultaneous tracking for assessment and comparison of locomotion. Individuals exposed to experimental solutions and control were placed in white plastic bowls (diameter 95 mm; height 30 mm) pre-filled with 50 ml of water solution specific to a given experimental group (M1; M2; MOA1; MOA2). Locomotion was recorded using a Sony HDR-CX240E (Sony, Japan) video camera and subsequently analysed by EthoVision XT 13 software (Noldus Information Technology, Wageningen, Netherlands). A multiple-arenas module recorded patterns of crayfish movement in all arenas for 1 h. Distance moved (cm), walking speed (cm/s), and activity (%) (percent of time spent in locomotion) were determined.

### Oxidative stress and antioxidant biomarkers

At the conclusion of the trial, the crayfish whole body was homogenized and prepared for evaluation of oxidative stress and antioxidant biomarkers following Stara *et al*.^25^. Biomarkers were measured spectrophotometrically (Infinite M200, Switzerland). Lipid peroxidation (LPO) measured as thiobarbituric acid reactive species (TBARS) was estimated by the method of Lushchak *et al*.^26^; total superoxide dismutase (SOD) activity according to Marklund and Marklund^27^; glutathione reductase (GR) activity according to Carlberg and Mannervik^28^; catalase (CAT) activity according to Beers and Sizer^29^; glutathione S-transferase (GST) activity according to Habig *et al*.^30^ and reduced glutathione (GSH) level following Tipple and Rogers^31^. Proteins were determined using the method of Bradford^32^.

### Histology

After recording of locomotion, six crayfish from each group were sacrificed on ice and routine histological procedures were carried out following Velisek *et al*.^33^. Briefly, crayfish were fixed in 10% buffered formalin. After 24 h, crayfish were decalcified for 4 h (slow decalcifier DC1 containing formic acid and formaldehyde; Labonord SAS, Germany), and the samples were embedded in the tissue processor Histomaster 2052/1.5 (MDS-group, Germany). Embedded crayfish were circumfused with paraffin, and sections from paraffin blocks were cut on a rotary microtome (5 μm) and stained with haematoxylin-eosin in an automatic slide staining system (TISSUE-TEK DRS 2000, SEKURA). Hepatopancreas and gill were examined by light microscopy and photographed with an E-600 camera (Olympus BX51, Japan). Histomorphological structures were evaluated in accordance with Ceccaldi^34^. Histological alterations were scored as (−) no histopathology; (+) histopathology in 20% of the fields; (++) histopathology in 20–60% of the fields, and (+++) histopathology in 60% of the fields.

### Statistical analysis

Differences in cumulative mortality between experimental groups and control were assessed using contingency tables (χ^2^). Kolmogorov-Smirnov and Bartlett’s tests were applied to assess normal distribution of data and the homoscedasticity of variance, respectively. ANOVA a Tukey HSD test was used for data with normal distribution. In case of non-normal distribution, a non-parametric Dunn test was performed. The significance level was set at *P* < 0.05.

## Results

### Survival and growth

The survival a growth of all group is presented in Table 2. Significant (H = 9.83, *P* = 0.007) differences from control in total mortality were found in crayfish exposed to both tested concentrations of metazachlor and metazachlor OA. Cumulative mortality in the M1 and M2 was 42.5 and 50.0%, respectively, while mortality of control was 9.5%. Mortality of crayfish in group MOA1 and MOA2 was 47.5 and 67.5%, respectively.

**Table 2:** Growth indices and cumulative mortality of marbled crayfish *Procambarus virginalis* early life stages in control and metazachlor- and metazachlor OA-exposed groups after 40 days.

Group	Control	M1	M2	MOA1	MOA1
Concentration	0	Metazachlor (0.0115 µmol/l)	Metazachlor (0.0790 µmol/l)	Metazachlor OA (0.0117 µmol/l)	Metazachlor OA (0.0805 µmol/l)
M_5_ (Mean ± SD, mg)	8.74 ± 1.01^a^	7.74 ± 1.29^a^	7.62 ± 0.90^a^	7.54 ± 0.83^a^	7.01 ± 0.60^b^
M_40_ (Mean ± SD, mg)	52.12 ± 8.14^a^	42.43 ± 5.83^b^	38.90 ± 4.41^b^	37.02 ± 5.54^b^	26.32 ± 3.08^c^
SGR	5.14 ± 0.15	4.83 ± 0.21	4.69 ± 0.19	4.54 ± 0.21	3.77 ± 0.11
I (%)	—	6.03 ± 0.45	8.75 ± 0.36	11.67 ± 0.87	26.65 ± 1.03
Total mortality (%)	9.50^a^	42.50^b^	50.00^b^	47.50^b^	67.50^c^

M5, M40 = Mean crayfish mass after 5 and 40 days exposure; SGR = mean specific growth rate in selected groups after 40 days exposure; I = inhibition of specific growth in exposed groups after 40 days exposure; SD = standard deviation. Different superscripts indicate significant (*P* < 0.05) differences.

**Figure 1:**
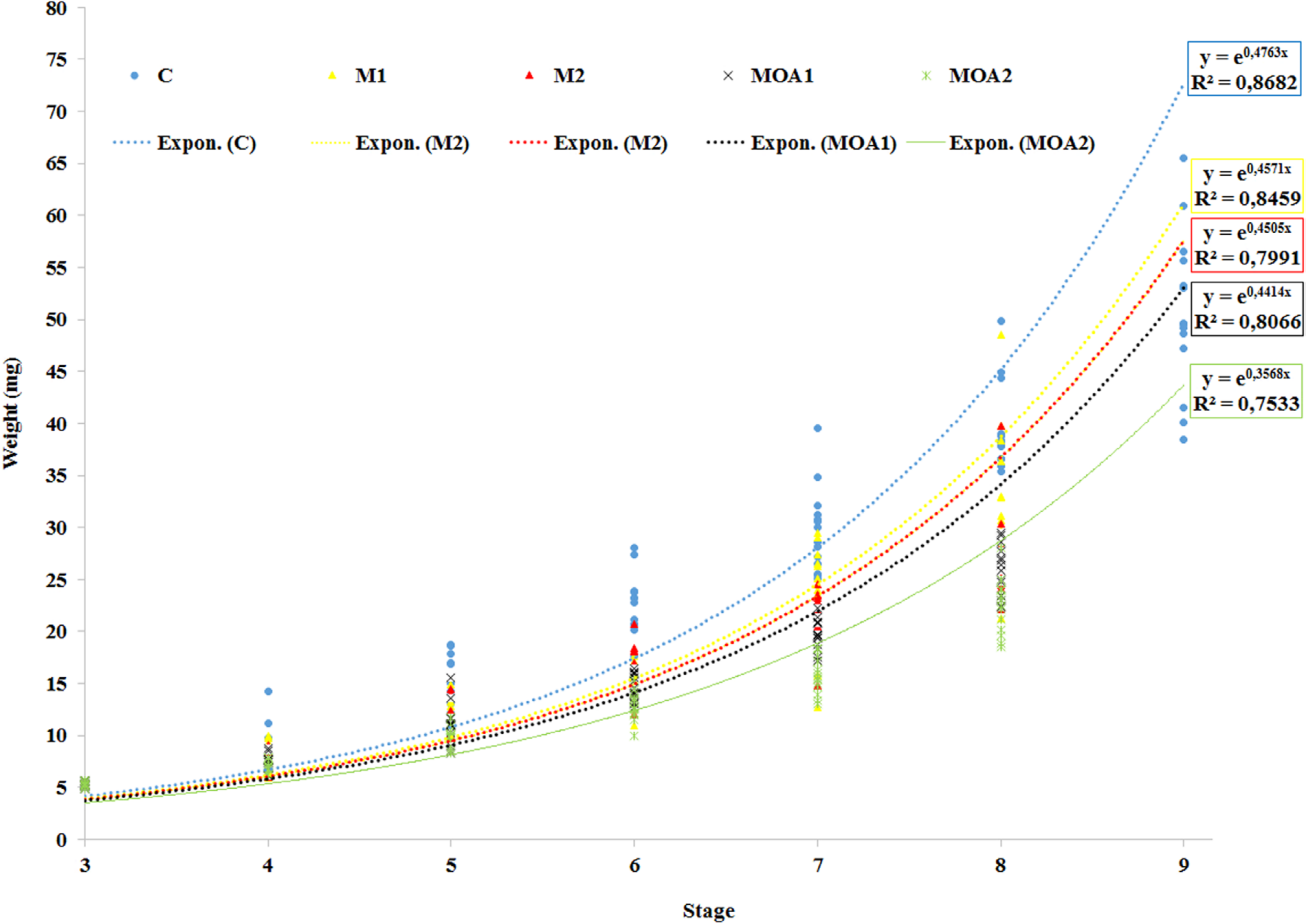
Body weight of early life stages of marbled crayfish *Procambarus virginalis* control, metazachlor (M1-0.0115 μmol/l, M2-0.0790 μmol/l), and metazachlor OA (MOA1-0.0117 μmol/l, MOA2-0.0805 μmol/l) exposure, during a 40-day toxicity test.

Mean growth relative to development stage of marbled crayfish during the trial is shown in Fig. 1. At stage 4, the MOA1 group showed significantly (H = 9.58, *P* = 0.009) lower weight than controls. At stage 5, all exposed groups exhibited significantly (H = 9.71, *P* = 0.008) lower weight than controls. Compared with control, inhibition of growth was 6.03, 8.75, 11.67, and 26.65% in M1, M2, MOA1, and MOA2, respectively.

### Early ontogeny and morphological anomalies

**Figure 2:**
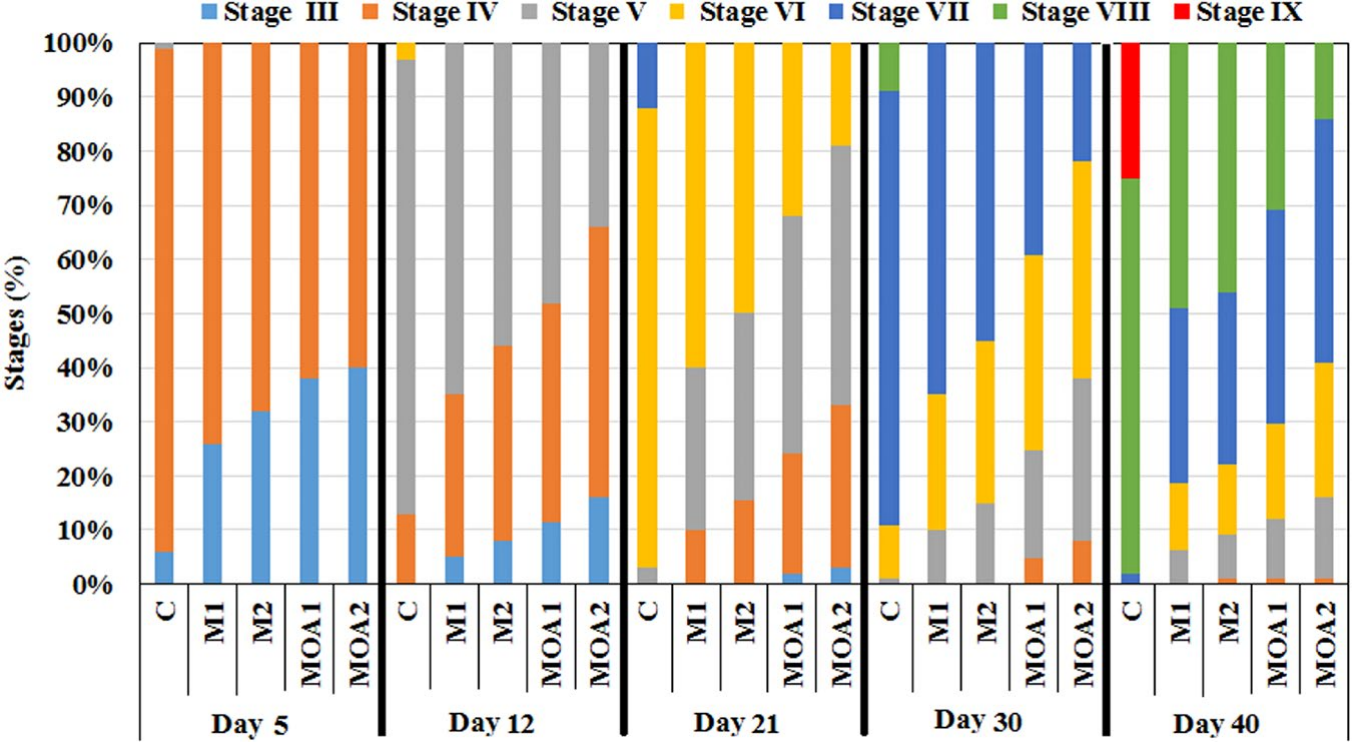
Marbled crayfish *Procambarus virginalis* developmental stages in control group and groups exposed to metazachlor (M1-0.0115 μmol/l, M2-0.0790 μmol/l) and metazachlor OA (MOA1-0.0117 μmol/l, MOA2-0.0805 μmol/l) during 40 days toxicity test.

Crayfish in metazachlor- and metazachlor OA- exposed groups showed significant (H = 9.56, *P* = 0.009) delayed ontogeny compared to the control (Fig. 2). At the end of the trial, 47, 46, 31, and 14% of individuals in M1, M2, MOA1, and MOA2 exposures, respectively, were at stage 8. Among controls, 73% were at stage 8 and 25% at stage 9. Morphological anomalies in early life stages were not observed in any group.

### Locomotion

**Figure 3:**
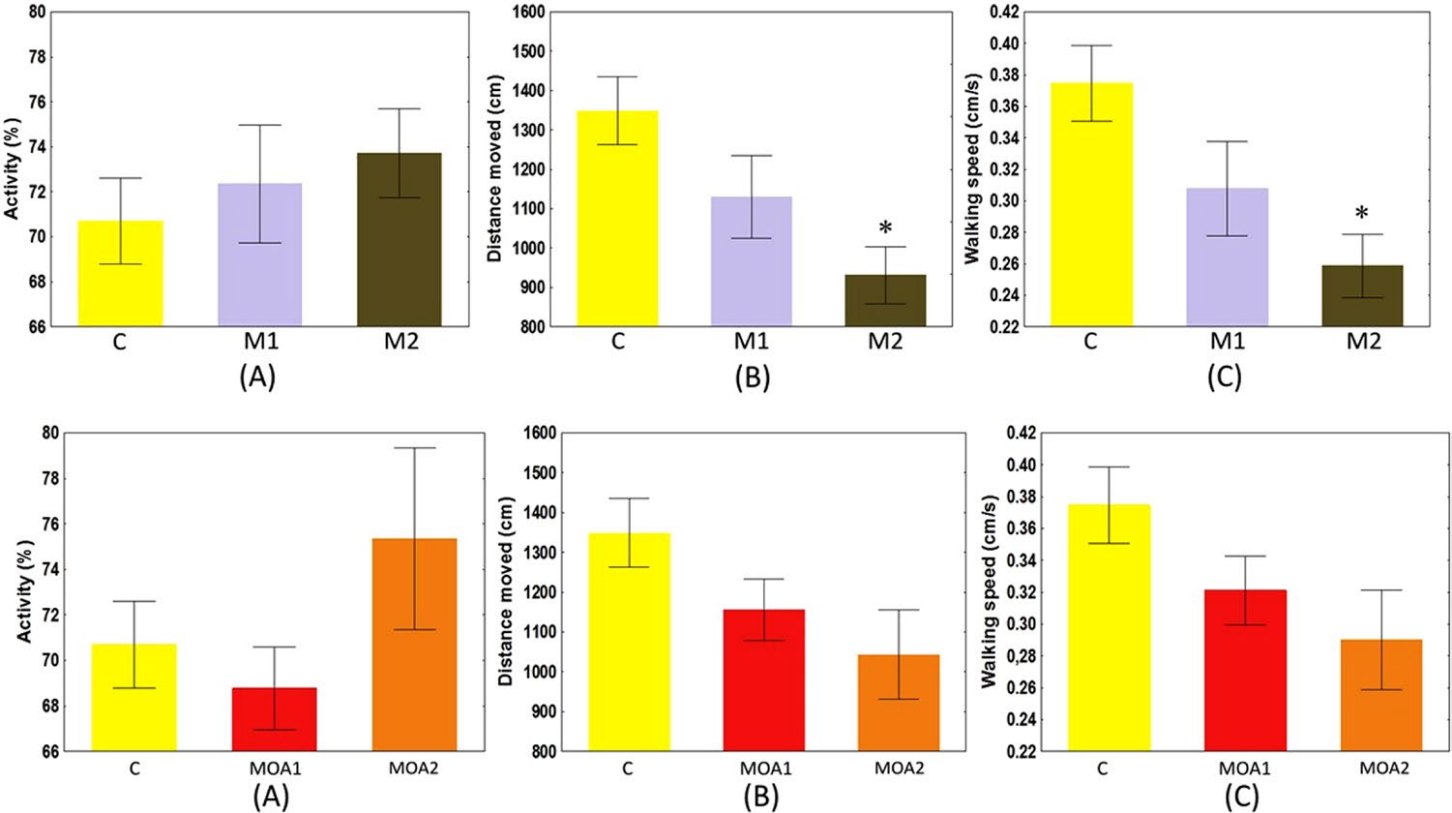
Mean activity level (**A**), mean total distance moved (**B**) and mean walking speed (**C**) of marbled crayfish *Procambarus virginalis* of control and exposed to metazachlor (M1-0.0115 μmol/l, M2-0.0790 μmol/l) and metazachlor OA (MOA1-0.0117 μmol/l, MOA2-0.0805 μmol/l). Data are mean ± SE.

No significant differences from controls were observed in activity level of metazachlor- and metazachlor OA-exposed crayfish (H = 0.98, *P* = 0.614) (Fig. 3A). Total distance moved (H = 9.68, *P* = 0.008) and walking speed (H = 9.76, *P* = 0.008) of the M2 group differed significantly from M1 and control. The M1 crayfish showed lower distance moved and speed than observed in controls, but values did not differ significantly (Fig. 3B,C). The MOA1 and MOA2 crayfish showed no significant differences from controls in evaluated parameters: distance moved (H = 4.32, *P* = 0.115), walking speed (H = 4.32, *P* = 0.115), and % activity (H = 4.86, *P* = 0.088).

### Oxidative stress and antioxidant response

The M1 group did not show significant differences from control in antioxidant CAT (H = 4.55, *P* = 0.095), SOD (H = 4.28, *P* = 0.121), GR (H = 4.26, *P* = 0.101), GST (H = 1.25, *P* = 0.354), GSH (H = 2.52, *P* = 0.241) or oxidative parameters (LPO) (H = 4.35, *P* = 0.119). Significantly lower SOD (H = 9.86, *P* = 0.004), CAT (H = 9.74, *P* = 0.008), GR (H = 9.54, *P* = 0.006), GST (H = 9.32, *P* = 0.008), and GSH (H = 8.77, *P* = 0.003) activity was observed in M2, MOA1, and MOA2 compared to control and M1 groups (Table 3). No significant differences (H = 3.01, *P* = 0.214) were seen in TBARS activity among groups.

**Table 3:** Antioxidant and oxidative parameters of whole-body homogenates of juvenile marbled crayfish *Procambarus virginali*s in control and metazachlor- and metazachlor OA-exposed groups after 40 days.

Group	Control	M1 Metazachlor (0.0115 µmol/l)	M2 Metazachlor (0.0790 µmol/l)	MOA1 Metazachlor OA (0.0117 µmol/l)	MOA2 Metazachlor OA (0.0805 µmol/l)
TBARS (nmol/mg protein),	0.29 ± 0.01^a^	0.28 ± 0.16^a^	0.31 ± 0.03^a^	0.29 ± 0.11^a^	0.28 ± 0.21^a^
SOD (nmol NBT/min/mg protein)	0.13 ± 0.02^a^	0.12 ± 0.02^a^	0.083 ± 0.01^b^	0.080 ± 0.01^b^	0.06 ± 0.01^b^
CAT (µmol H_2_O_2_/min/mg protein)	0.32 ± 0.13^a^	0.30 ± 0.12^a^	0.20 ± 0.11^b^	0.19 ± 0.15^b^	0.17 ± 0.12^b^
GR (nmol NADPH/min/mg protein)	0.17 ± 0.22^a^	0.16 ± 0.12^a^	0.09 ± 0.05^b^	0.07 ± 0.06^b^	0.07 ± 0.01^b^
GST (nmol/min/mg protein)	4.22 ± 1.22^a^	3.97 ± 1.29^a^	2.00 ± 0.91^b^	1.91 ± 1.32^b^	1.81 ± 1.72^b^
GSH (nmol GSH/mg protein)	38.26 ± 7.21^a^	40.22 ± 9.32^a^	32.05 ± 3.13^b^	30.89 ± 3.55^b^	29.33 ± 4.78^b^

Data are mean ± SD, n = 6. Different superscripts indicate significant (*P* < 0.05) differences.

### Histology

**Figure 4:**
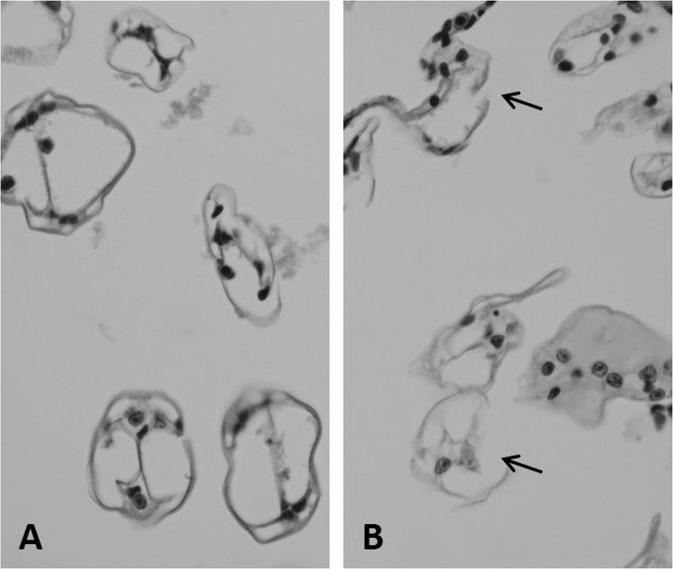
Marbled crayfish *Procambarus virginalis* gill: (**A**) control with typical structure of gill lamellae; (**B**) MOA2 exposed to 0.0805 μmol/l metazachlor OA; arrows show disintegration of lamellar structure; × 400; H&E.

Gill of control and M1 groups showed typical histological structure, while slight abnormalities ranging from wall thinning to focal disintegration of branchial structure were observed in the M2 and the metazachlor OA-exposed groups (Fig. 4). Described anomalies were more frequent in the M2 (+++) and MOA2 (+++) groups.

There was no pathology observed in hepatopancreas of exposed groups or controls. The structure of tubules appeared physiologically normal with the presence of all cell structure types. Differences from controls in the proportion of individual cell types were observed in the M2 (++) and MOA2 (+++) groups, with higher numbers of large univacuolar cells.

## Discussion

Decapods can serve as an excellent model species to increase the ecotoxicological knowledge base^33,35–37^. They are large and easily reared in the laboratory, making them useful not only for toxicity testing, but as an invertebrate model for disciplines including genetics, development, and cell biology^35^. This study is the first evaluating the effects of metazachlor and its major metabolite metazachlor OA at relevant environmental concentrations on early life stages of an invertebrate.

For the test we used parthenogenetic juveniles (stage 3 of embryonic development) offspring of a single marbled crayfish female. Marbled crayfish is also valuable as a model organism, due to its biological characteristics and production of genetically identical offspring. The marbled crayfish is unique among decapods by reproduction via obligatory apomictic parthenogenesis, and males are unknown^23,35^, Despite this genetic uniformity, substantial variability in growth rate, age at maturation, fecundity and colour patterns exist. These variations have not been adequately explored and represent a wide field for research. Marbled crayfish locomotion is significantly affected by drug exposure. And can be used as suitable model for the investigation of mechanisms of physiological response after drug exposure^38^. Information on the sensitivity of marbled crayfish to chemicals is scarce, but it seems to be a suitable model organism for the evaluation of responses to pesticides and drugs, due to its availability and genetic uniformity^35^. The marbled crayfish is used in ecotoxicological studies^33,36,37,39^, and it can be expected that its exploitation for this purpose will increase.

Significant differences from control were found in mortality of crayfish exposed to both concentrations of tested compounds. Cumulative mortality in the M1 and M2 (metazachlor) was 42.5, and 50.0%, respectively. Mortality in the MOA1 and MOA2 (metazachlor OA) groups was 47.5, and 67.5%, respectively. Early life stages often show higher mortality than observed in adults following exposure to xenobiotics^40–43^. The early life stages of marbled crayfish are reported to be more sensitive than fish (96hLC50 4.0–15 mg/l) and *Daphnia magna* (48hEC50 22.3 mg/l) to metazachlor^6^. Metazachlor and metazachlor OA have been recorded in surface water at concentrations ranging from 0.1–100 μg/^10,11,13,19^ and 0.1–3.2 μg/l^19^, respectively. Our results suggest that environmental concentrations of this herbicide and its major metabolite in surface waters may be lethal for early life stages of crayfish.

Metazachlor- and metazachlor OA-exposed crayfish showed lower growth in early life stages. Compared to the control, observed inhibition of growth was 6.03, 8.75, 11.67, and 26.65% in groups M1, M2, MOA1, and MOA2, respectively (Table 2). Reduction in growth may be the result of diversion of energy to detoxification and the antioxidant systems as well as to delay of early ontogenetic development. Retarded growth of early life stages of crayfish has also been reported after triazine^41^, chloroacetanilide^37^ and triazine metabolite^33,44^ exposure.

Research indicates that early ontogenetic development is a sensitive parameter for evaluating the effects of herbicides on fish^45–48^ and crayfish^36,37,44^. In the present study, crayfish exposed to metazachlor and metazachlor OA at both tested concentrations showed significant delay in development.

Studies of chronic exposure to herbicides or other pollutants often use sophisticated observations such as grazing and exploration behaviour^49,50^. We previously investigated basic behaviour patterns such as activity level and locomotion^37^. In the present study, we observed possible impairment of sensory-mediated behaviour resulting in a concentration-dependent decreased pattern of movement. Significant differences were observed in distance moved and speed, but not in activity (Fig. 3). This suggests the potential of metazachlor exposure to result in slower cue responses. Crayfish are heavily dependent on processing a wide spectrum of visual, chemical, and water-borne signals^51^ with escape behaviour as an appropriate response. A delayed response to predator presence and slower locomotion can be fatal. Metazachlor OA-exposed crayfish exhibited behaviour patterns similar to the metazachlor groups. Reduced locomotion could be detrimental to early stages of crayfish.

Xenobiotics can affect physiological and biochemical status, leading to the production of reactive oxygen species (ROS)^25,52^. The most important antioxidant biomarkers regulating ROS are SOD, CAT, GPx, GSH, GR, and GST^53,54^. Levels of antioxidant biomarkers in fish depend on the concentration of the xenobiotic and exposure duration^55,56^. The M2, MOA1, and MOA2-exposed crayfish showed significant reduction in SOD, CAT, GR, GST, and GSH levels, apparently maintaining cells in the equilibrium state, combating ROS production, since oxidative damage (LPO) was not observed. The reduced antioxidant levels in exposed crayfish were related to detoxification of oxidative stress, suggesting that the tested substances affect antioxidant defence systems. A similar trend to reduced antioxidant biomarker levels not accompanied by LPO has been reported in decapods after pesticide exposure^33,36,37,57–60^. These effects have also been confirmed in fish after triazine exposure^54^.

Gill tissue condition is generally considered a good indicator of water quality and appropriate for evaluation of environmental impact^61^. Damage to the epithelial layer and disintegration of branchial structures increase the diffusion distance between water and blood, affecting respiration and ionic regulation by inhibiting key transport processes^61,62^. Velisek *et al*.^37^ found changes in marbled crayfish gill including focal haemocytic infiltration with enlargement of the intra-lamellar space packed with granular substance after metolachlor OA (42 and 420 μg/l) exposure. Crayfish hepatopancreas is involved in absorption and storage of nutrients and can synthesize digestive enzymes for food digestion. It is the primary organ of biotransformation and detoxification in crustaceans. In the present study, the hepatopancreas showed differences from controls in the proportion of cell types, especially higher numbers of B cells and lower numbers of R cells in M2 and MOA2 groups. The B cells are involved in the absorption of nutrients from the hepatopancreatic tubular lumen and are responsible for intracellular digestion, concentrating absorbed materials in the large vacuole. The function of R cells lies in storage of small quantities of lipids and other surplus materials such as glycoproteins as energy reserves^63–65^. Velisek *et al*.^36^ found alteration of the tubular system of hepatopancreas including focal disintegration of tubular epithelium and reduction in epithelial cell numbers, especially B-cells, in marbled crayfish after exposure to s-metolachlor at 110 μg/l. Conversely, Stara *et al*.^58^ found no changes in hepatopancreas of red swamp crayfish (*Procambarus clarkii*) after prometryn (0.51–1440 μg/l) exposure. Histological anomalies of hepatopancreas in the present study reflect increased demand for digestion of nutrients together with depletion of stored material, indicating impaired nutritional status.

To conclude, antioxidant levels and early ontogeny of marbled crayfish are useful biomarkers for monitoring aquatic environments contaminated by the herbicide metazachlor and its metabolite, metazachlor OA. Differences in mortality, growth, early ontogenesis, antioxidant systems, and behaviour, as well as minimal alterations in gill were observed following exposure to environmentally relevant concentrations of tested compounds. Metazachlor OA showed a more pronounced effect on physiology of early life stages of marbled crayfish than did its parent substance metazachlor. Further research will be needed before *Procambarus virginalis* can be established as a bioindicator for monitoring aquatic environments for chloroacetamide herbicides.
